# Patient perspectives on the development of a prescription opioid use disorder

**DOI:** 10.1192/j.eurpsy.2025.93

**Published:** 2025-08-26

**Authors:** L. E. M. Davies, A. Schellekens

**Affiliations:** 1 Utrecht University, Utrecht; 2 Department of Psychiatry, Donders Institute for Brain, Cognition and Behaviour, Radboud University Medical Center; 3 Nijmegen Institute for Scientist Practitioners in Addiction, Nijmegen, Netherlands

## Abstract

**Introduction:**

In the past decade, prescription opioid use increased exponentially and concomitantly prescription opioid use disorders (OUD) are becoming more common. While substantial research has identified clinical risk factors, little attention has been paid to the lived experiences that contribute to the development of OUD.

**Objectives:**

This study aimed to explore and document patients’ experiences on how they developed a prescription OUD.

**Methods:**

We conducted in-depth, semi-structured interviews with 25 adults with chronic non-cancer pain currently undergoing treatment for prescription OUD. The interviews explored their experiences with long-term opioid use, attitudes toward opioids, and access to prescriptions. Transcripts were analysed using directed content analysis to identify recurring themes.

**Results:**

Participants identified three key themes influencing the development of OUD: (1) experiences driving initiation, (2) experiences driving continuation, and (3) experiences with prescription OUD. Beyond pain management, factors such as patient-provider communication, care coordination, provider vigilance, and environmental support significantly shaped opioid use patterns.Participants cited a lack of guidance during both initial and long-term opioid use, easy access to prescriptions, and insufficient monitoring as major contributors to OUD. Poorly controlled pain and high levels of stress were also highlighted as critical drivers of continued opioid use.

**Conclusions:**

Patients described a distinctive pathway to prescription OUD, contrasting with other substance use disorders, with negative reinforcement playing a particularly prominent role in the early stages of opioid use. Their perspectives reveal critical gaps in guidance and monitoring during opioid therapy, highlighting opportunities for intervention and improvement.

This talk will explore how these insights can inform prevention strategies, improve care coordination, and support better outcomes for patients at risk of OUD.

**Disclosure of Interest:**

None Declared

